# Risk factors for migraine disease progression: a narrative review for a patient-centered approach

**DOI:** 10.1007/s00415-023-11880-2

**Published:** 2023-08-24

**Authors:** Richard B. Lipton, Dawn C. Buse, Stephanie J. Nahas, Gretchen E. Tietjen, Vincent T. Martin, Elin Löf, Thomas Brevig, Roger Cady, Hans-Christoph Diener

**Affiliations:** 1 Department of Neurology, Albert Einstein College of Medicine, Bronx, NY USA; 2 Vector Psychometric Group, LLC, Chapel Hill, NC USA; 3 Department of Neurology, Thomas Jefferson University, Jefferson Headache Center, Philadelphia, PA USA; 4 University of Toledo College of Medicine and Life Sciences, Toledo, OH USA; 5 University of Cincinnati Headache and Facial Pain Center, Cincinnati, OH USA; 6 H. Lundbeck A/S, Copenhagen, Denmark; 7 Lundbeck LLC, Deerfield, IL USA; 8 RK Consults, Ozark, MO USA; 9 Missouri State University, Springfield, MO USA; 10 Institute for Medical Informatics, Biometry and Epidemiology, Medical Faculty, University Duisburg-Essen, Essen, Germany

**Keywords:** Migraine progression, Chronic migraine, Episodic migraine, Risk factors, Prevention strategies

## Abstract

**Background:**

In individuals with migraine, attacks may increase in frequency, severity, or both. Preventing migraine progression has emerged as a treatment goal in headache subspecialty practice, but there may be less awareness in general neurology or primary care settings where most people with migraine who seek treatment consult. Herein, we review the definition of and risk factors for migraine progression and consider strategies that could reduce its risk.

**Methods:**

A group of headache expert healthcare professionals, clinicians, and researchers reviewed published evidence documenting factors associated with increased or decreased rates of migraine progression and established expert opinions for disease management recommendations. Strength of evidence was rated as good, moderate, or based solely on expert opinion, using modified criteria for causation developed by AB Hill.

**Results:**

Migraine progression is commonly operationally defined as the transition from ≤ 15 to ≥ 15 monthly headache days among people with migraine; however, this does not necessarily constitute a fundamental change in migraine biology and other definitions should be considered. Established and theoretical key risk factors for migraine progression were categorized into five domains: migraine disease characteristics, treatment-related factors, comorbidities, lifestyle/exogenous factors, and demographic factors. Within these domains, good evidence supports the following risk factors: poorly optimized acute headache treatment, cutaneous allodynia, acute medication overuse, selected psychiatric symptoms, extra-cephalic chronic pain conditions, metabolism-related comorbidities, sleep disturbances, respiratory conditions, former/current high caffeine intake, physical inactivity, financial constraints, tobacco use, and personal triggers as risk factors. Protective actions that may mitigate migraine progression are sparsely investigated in published literature; our discussion of these factors is primarily based on expert opinion.

**Conclusions:**

Recognizing risk factors for migraine progression will allow healthcare providers to suggest protective actions against migraine progression (Supplementary Fig. 1). Intervention studies are needed to weight the risk factors and test the clinical benefit of hypothesized mitigation strategies that emerge from epidemiological evidence.

**Supplementary Information:**

The online version contains supplementary material available at 10.1007/s00415-023-11880-2.

## Introduction

Migraine is a debilitating chronic neurologic disorder associated with substantial personal, economic, and societal burdens and reductions in health-related quality of life (HRQoL) [1, 2]. Among adults, the prevalence of migraine is approximately 14%, peaking in midlife, with a substantial female preponderance [3]. Roughly one-third of people with migraine experience ≥ 4 monthly headache days (MHDs) and about 7% have ≥ 15 MHDs [4]. The traditional goals of preventive headache treatments are to reduce attack frequency and severity, while acute therapy is intended to relieve pain and restore function when attacks occur; recent papers have highlighted the potential of preventing disease progression [5, 6].

The clinical course of migraine is heterogeneous, with frequency, severity, symptom profiles, and associated disability varying over time among individuals [7–10]. However, some individuals will experience sustained worsening (i.e., progression) of their disease state. Clinicians who treat migraine should understand the phenomenon of migraine disease progression, as well as the potentially modifiable risk factors, and protective actions that influence the course of disease. Given the size of the migraine population and the large percentage of patients seeking medical attention in the primary care setting, primary care providers and other clinician specialists, such as neurologists, must be equipped to manage migraine [11]. Results from OVERCOME (ObserVational survey of the Epidemiology, tReatment, and Care Of MigrainE), conducted in individuals with migraine in the United States, indicated that the over the course of their lifetimes, most people with migraine who seek medical care do so in primary care (70.3%) while only 15.6% see neurologists or headache specialists [11]. When assessing a patient’s risk profile for migraine progression, it is important to include the patient as a partner in developing a management approach. Clinicians should identify factors that are modifiable and most likely to improve the patient’s quality of life. Treatment success should be defined as a series of milestones, each of which becomes a foundation for the next step towards optimal treatment.

Based on evidence reviewed herein, we believe that preventing progression should be added to the list of therapeutic goals, not just for specialists, but in the primary care settings where most people with migraine receive their medical care. The objective of this review is to provide clinicians with the tools to understand migraine disease progression, to identify its risk factors, and to suggest potential mitigation strategies to prevent progression that are supported by research and expert opinion.

## Methods

An advisory board of seven headache expert healthcare professionals from the United States (RBL, DCB, SJN, GET, VTM, RC) and Germany (HCD) was convened to conduct a narrative review including gathering and evaluating published evidence regarding risk factors for the progression of migraine disease and developing recommendations for practicing clinicians that may help prevent disease progression. The definition of migraine disease progression used here is detailed below. Publications for review were selected from a PubMed search using keywords relevant to migraine disease progression, disease progression risk factors, and protective actions against migraine disease progression. Using AB Hill’s criteria for causation [12], risk factors and protective actions were classified into three categories based on the strength of evidence: (1) good evidence (supported by ≥ 2 studies with ≥ 100 participants performed at multiple centers, by ≥ 2 studies that surveyed a large geographic area, or by a systematic review with meta-analysis); (2) moderate evidence (≥ 1 peer-reviewed study); and (3) expert opinion (lack of peer-reviewed study, but based on professional experience of the advisory board). Professional experience and opinion are considered valuable when high-quality evidence is not available [13]. A similar approach to review risk factors for progression was used in a previous report [14].

## Defining disease progression in migraine

The most common definition of migraine disease progression is an increase in MHD frequency from < 15 MHDs to ≥ 15 MHDs among those who meet criteria for migraine; this process is also referred to as “chronification” or “transformation” [15–17]. This definition is commonly used in clinical trials as a standard metric to describe migraine progression. The transition from < 15 MHDs to ≥ 15 MHDs aligns with the International Classification of Headache Disorders, 3rd edition (ICHD-3) definition of chronic migraine (CM). ICHD-3 criteria characterize CM by the occurrence of headache on ≥ 15 days/month (≥ 15 MHDs) for > 3 months, with ≥ 8 days/month exhibiting migraine features or responding to triptan or ergot medications [17]. The complement to CM is episodic migraine (EM), with < 15 MHDs [18]. Though EM is not a formal diagnosis in the ICHD-3, this oversight was acknowledged and addressed by the current chair of the classification committee, who offered an interim definition of EM as headache occurring on less than 15 days per month over the last 3 months, including some headache days meeting criteria for migraine [19].

In studies examining the EM–CM transition [8–10], the estimated annual progression rate was approximately 3% in population-based studies that sampled annually [20, 21]. In clinic-based studies, annual transition rates were as high as 14% [22]. CM onset occurs at higher rates in particular subgroups, and transition between EM and CM may be bidirectional [7–9, 20, 23, 24]. The population-based Chronic Migraine Epidemiology and Outcomes (CaMEO) study conducted surveys at 3-month intervals and showed that, over 1 year of follow-up, more than 7% of those with EM at baseline had at least one 3-month period when they met the criteria for CM, while almost three-quarters of those with CM at baseline had at least one 3-month period when they did not meet the ≥ 15 MHDs criterion [7]. In other words, transitions from EM to CM and from CM to EM are common among people with migraine.

Crossing the 15-MHD threshold, however, does not necessarily constitute a fundamental change in the biology of the underlying disease. For example, an increase from 14 to 16 MHDs may be a minor natural fluctuation, whereas an increase from 1 to 14 MHDs or from 15 to 28 MHDs—neither of which crosses the 15-MHD threshold—could represent a major change in illness. A growing body of evidence and opinion suggests that the 15-day chronification/transformation threshold to distinguish between EM and CM may need to be reconsidered because it does not capture the substantial differences in disability across the full spectrum of headache frequency, nor does it reflect the treatment needs of patients [25]. Persons with high-frequency EM (8–14 MHDs) are more similar in symptomology and burden to those with low-frequency CM (15–22 MHDs) than to those with low-frequency EM (0–3 MHDs) or medium-frequency EM (4–7 MHDs). Several studies suggest that persons with high-frequency EM may be undergoing disease progression, without having crossed the threshold which defined CM [4, 25]. In addition, the optimal definition of progression has been questioned. A 5-day-per-month increase in frequency from baseline that is sustained for ≥ 3 months may better reflect disease worsening than traversing an arbitrary value, such as 15 MHDs [26]. A sustained increase is noteworthy because there can be natural transient fluctuations in headache frequency related to environmental factors (e.g., fluctuations in weather and seasonal allergens), stressful life events, or many other individual or combinations of factors—or for no discernible reason—as seen in data collected every 3 months from the CaMEO study [7].

The previously mentioned limitations of using a 15-MHD threshold suggests that alternative or additional measures may be useful in identifying migraine progression. For example, subgroups defined by a broader range of attributes, such as the burden of migraine symptoms, average migraine pain, headache-related disability, ictal or interictal cutaneous allodynia (experience of pain or discomfort from ordinarily nonpainful stimuli), acute headache medication use, and presence and severity of comorbidities (such as anxiety and depression) in addition to MHDs may be clinically helpful [27, 28]. Alternatively, patient-reported outcome measures such as the Migraine Treatment Optimization Questionnaire (mTOQ; measures acute treatment optimization), the Patient Global Impression of Change, and the Migraine Disability Assessment Scale (MIDAS), in addition to rate of increase in MHDs and the use of acute headache medication may be more appropriate criteria for identifying persons at risk for progression [29–31]. Much of the available literature on migraine progression relies primarily on the transition from EM to CM and is, therefore, subject to the limitations outlined above.

Medication overuse headache (MOH) is a secondary headache disorder occurring on 15 or more days/month in patients with a pre-existing primary headache; it develops as a consequence of regular overuse of acute or symptomatic headache medication (on 10 or more or 15 or more days/month, depending on the medication) for more than 3 months [17]. Overuse of acute medication is a risk factor for progression but may also be a marker of the process of progression [32, 33]. Increased acute medication use and overuse can be a cause of or a consequence of headache progression [10]. Estimates of MOH prevalence range from 0.5 to 2.6% in the general population but is much more common in the patient population with CM, where rates of 11–70% have been reported [9, 34].

Instead of examining CM as outcome, several older studies have examined chronic daily headache (CDH). CDH is a condition defined by headache on 15 or more days per month. In CDH of long duration (characterized by headaches that last 4 or more hours per day) the differential diagnosis includes chronic migraine, chronic tension-type headache, new daily persistent headache, and hemicrania continua [35]. Most people who develop CDH in the setting of antecedent migraine meet the case definition for CM, but some studies did not have sufficient detail to assign this diagnosis. In the review that follows, we indicate whether the outcome in the study was CM or CDH.

The underlying pathophysiological mechanisms associated with migraine progression are not yet completely understood. However, recent evidence suggests that progression is associated with both structural and functional alterations in the brain, influenced by genetic and environmental factors [9, 14, 36]. As migraine progresses, frequent and sustained activation of the trigeminovascular system and its central connections may lead to neuroplastic changes and a lowering of the threshold for initiating migraine attacks [21]. Thus, migraine progression may be part of a cyclic process [21]. This hypothesis is supported by manifestations of peripheral and central sensitization such as the development of allodynia during migraine attacks, emergence of allodynia between migraine attacks (interictally), and increased use of acute headache medications. Increasing headache frequency could be a consequence of increased sensitivity to triggers. Central sensitization is associated with spontaneous firing of the second-order neurons in the trigeminocervical complex and a reduced threshold for activation of peripheral and central nociceptive pathways [18], giving rise to allodynia [37]. Animal studies have demonstrated reductions in thresholds to pain triggers following repeated administration with acute pain medications [38, 39]. Increased use of acute headache medications that lead to central sensitization is associated with decreased effectiveness of the acute headache medication [39–42], resulting in a vicious cycle of an increased number of headache days requiring excessive amounts of acute headache medication. Without proper migraine management, this may lead to the development of CM and MOH.

## Risk factors for migraine disease progression

Numerous factors have been associated with progression from EM to CM (or CDH) [14, 43]. In addition, factors associated with the onset of MOH have also been identified [34]. The strength of evidence is greater for some factors than for others, although this may reflect the strength of the methodology, study design, and sample size of the studies performed, and not necessarily the strength of association. Further limitations include the inability to ascertain the directionality of associations in cross-sectional studies and the likelihood that there are additional risk factors that have yet to be identified or rigorously studied. Key factors found in this review were categorized broadly into 5 domains: (1) migraine disease characteristics (e.g., frequency, associated symptoms), (2) treatment-related, (3) comorbidities, (4) lifestyle/exogenous factors, and (5) demographic factors (Table 1) [14, 43]. Supplementary Table 1 is a printable version of Table 1.

**Table 1:** Summary of key risk factors of migraine disease progression and possible protective actions

	Risk factors	Evidence for the risk factor association with progression	Possible protective actions	Evidence that addressing risk factors reduces progression risk
Migraine disease characteristics	*Any migraine disease characteristic risk factor*		Track attack frequency, severity, and treatment use through a diary or app	O
			Optimize acute and preventive treatment using both pharmacologic and non-pharmacologic options [1, 95–101]	↑
			Educate patients on healthy lifestyle choices and how to implement change	O
	≥ 5 MHDs (moderate risk) or ≥ 10 MHDs (high risk) [43]	↔	Progressive muscle relaxation [115]	↔
	Cutaneous allodynia [45–47]	↑		
	Persistent, frequent nausea [48]	↔		
Suboptimal treatment	*Any treatment-related factor*		Use non-pharmacologic approaches such as neuromodulation and biobehavioral therapies [1, 95]	↔
			Change dose or route of administration	O
			Add or switch to another acute or preventive medication	O
			Add preventive therapies	O
			Optimize adherence	O
	Suboptimal acute treatment [21, 24, 49, 50, 102]	↑	Optimize acute migraine medication	O
			Educate patients about their acute migraine medication [103]	O
	Acute medication overuse [22, 47, 52, 91]	↑	Educate patients about appropriate exposure to acute migraine medication and risks of high use	O
			Limit acute medication use to < 8 days/month	O
	Preventive medication not satisfactorily effective/tolerated	O	Optimize preventive medication [5, 104–111]	↑
			Aim for treatment to reduce days of moderate or severe pain to < 4 days per month	O
			Take preventive medication as prescribed	O
			Consider adding non-pharmacologic preventive options	O
Comorbidities	*Any comorbidity*		Identify common comorbidities and educate patients on comorbid conditions	O
			Optimize assessment and treatment of comorbid conditions where appropriate	O
			Refer for treatment when appropriate	O
			Teach self-management skills and strategies when appropriate	O
	Psychiatric symptoms, especially depression and anxiety [27, 43, 66, 67]	↑	Assess, monitor, treat, or refer to mental health professionals for pharmacologic and/or behavioral treatment	O
	Other (non-headache) chronic pain conditions [54, 56, 57]	↑		
	Head and neck injury, TBI [68]	↔	Physical therapy	O
	Metabolism-related comorbidities (metabolic syndrome, insulin resistance, overweight/underweight) [44, 58–60]	↑	Exercise/physical activity [121]	O
			Diet [128]	O
			Optimized disease management	O
	Sleep disturbances, including insomnia, snoring, and restless leg syndrome [27, 44, 61–64]	↑	Healthy sleep hygiene and practices	O
			Targeted behavioral sleep intervention [122]	↔
			May need a sleep study (obstructive sleep apnea) [123]	↔
			May need CBTi (insomnia) [123]	↔
	Respiratory conditions such as allergic rhinitis and asthma [51, 65]	↑		
	Multiple other comorbidities [67]	↔		
Lifestyle and exogenous factors	*Any lifestyle or exogenous risk factor*		Self-management strategies and programs [125–127]	↔
	Stress, including adverse childhood experiences, stressful life events, previous assault [71–74]	↔	Refer for therapy and support	O
			Stress management, CBT, biofeedback, relaxation training, exercise, social support	O
			Foster resilience	O
	Poor nutrition [78, 79, 128]	↔	Eat regular, balanced meals	O
	Poor hydration [80]	↔	Stay hydrated with water and other hydrating liquids	O
			Provide access resources such as a note for work to allow more access to water	O
	Former and current high caffeine intake [54, 81–83]	↑	Reduce caffeine intake to no more than 2 caffeinated beverages a day	O
	Physical inactivity [54, 84, 129]	↑	Regular exercise/physical activity [121]	↔
	Poor sleep quality and duration [64]	↔	Healthy sleep hygiene and practices [122]	↔
	Smoking tobacco [54, 85]	↑	Tobacco cessation	O
	Exposure to personal triggers [64, 82, 86]	↑	Keep a headache diary to identify triggers [124]	↔
Demographic factors	Female sex [87]	↔		
	Hormonal status [88–90]	↔		
	Low level of education attainment [54]	↔		
	Ongoing financial constraints [43, 87, 91]	↑		

Level of evidence: ↑ = good evidence; ↔ = moderate evidence; O = expert opinion

*CBT* cognitive behavioral therapy, *CBTi* cognitive behavioral therapy for insomnia, *MHDs* monthly headache days, *TBI* traumatic brain injury

### Migraine disease characteristics

Headache frequency is one of the strongest risk factors for progression from EM to CM. In the longitudinal Frequent Headache Epidemiology Study, the risk of progression to CDH was positively correlated with baseline headache frequency, with a low risk for those with < 3 MHDs and rapidly increasing risk for those with higher frequencies [44]. A meta-analysis found that ≥ 10 MHDs was associated with an almost sixfold increased risk of progressing to CM [43].

A cross-sectional analysis of the longitudinal, population-based American Migraine Prevalence and Prevention (AMPP) study found that the prevalence of severe allodynia increased with headache frequency, disability, and duration, suggesting that allodynia and disease burden are linked, though directionality was not established [45]. The AMPP study also found that the prevalence of allodynia was higher in individuals with CM than in those with EM [46]. The prospective longitudinal Leiden University Migraine Neuro-Analysis (LUMINA) study found that cutaneous allodynia at baseline was an independent predictor of migraine-day frequency at follow-up (mean ± standard deviation: 93 ± 30 weeks post-baseline), with a > 11-fold increased risk of having an increased frequency of migraine days [47]. Cutaneous allodynia at baseline was also an independent predictor of the number of medication days at follow-up [47].

Nausea is a common migraine-associated symptom, occurring in approximately 70% of those with migraine [14]. Data from the AMPP study found that after controlling for socioeconomic factors, migraine symptom composite score, headache-related disability, depression, and opioid use, participants with frequent and persistent nausea (defined as nausea during at least half of episodes and persisting over a span of 2 years) were more than twice as likely to progress from EM to CM [48].

### Treatment-related factors

Suboptimal acute treatment of EM is associated with an increased risk of progression to CM [21]. Continued use of suboptimal acute treatment may lead to redosing or to use of a different acute medication, potentially increasing medication overuse and the risk of further disease progression [21, 49, 50].

An analysis of 5681 participants from the longitudinal AMPP study found that—after adjusting for sociodemographic factors, MHDs, headache-related disability, and acute medication use—lower levels of acute treatment optimization at baseline were associated with higher rates of progression from EM to CM at follow-up (1 year) [21]. These results have three potential interpretations. First, suboptimal treatment may lead to migraine disease progression, as suboptimal acute treatment is associated with persistent activation of central pain pathways and may contribute to the development of central sensitization and allodynia. Second, the association between poor acute treatment response and progression may result from confounding factor(s) (i.e., there is another variable influencing acute treatment response and progression that accounts for their association). For example, those with long and severe migraine attacks or rapid-onset migraine attacks may experience poor acute treatment results in general and may be at increased risk for progression. A third interpretation is that poor response to acute treatment may be a manifestation of the process that leads to progression. For example, progression may manifest as central sensitization, leading to reduced response to triptans. In a clinical trial, if improving acute treatment optimization reduced the rates of progression, that would support the first hypothesis.

Acute medication overuse in general is a risk factor for disease progression. A meta-analysis found that medication overuse was associated with an 8.8-fold increased risk of progressing to CM [43]. In the population-based AMPP study, the risk of new-onset CM at 1-year follow-up was 2.6-fold higher in those with medication overuse [51]. Medication overuse was associated with an increased risk of CM onset in a clinic-based study [22]. Another longitudinal study found that medication overuse was a significant predictor of CDH persistence in adolescents and adults [52].

Distinct acute medication types have different risk profiles for increasing migraine frequency and developing MOH [49, 50]. The AMPP study found that the risk of progression from EM to CM (1-year follow-up) was highest based on any exposure to barbiturate-containing medications (relative to acetaminophen: 73% increased odds), followed by opioids (44% increased odds) [24]. Nonsteroidal anti-inflammatory drugs (NSAIDs) were potentially protective against the transition from EM to CM at low to moderate MHD frequency (< 10 MHDs) but were associated with an increased risk of CM onset in those with 10–14 MHDs [24]. Another analysis of the AMPP data using 5-year follow-up data confirmed the protective effect of NSAIDs at lower MHD frequency and increased risk of CM with NSAIDs at higher MHD frequency [53]; the analysis also found that a 1-day increase in triptan use per month was associated with a 7% increase in the risk of CM onset. The longitudinal, population-based, cohort Nord-Trøndelag Health Study (HUNT) reported that use of tranquilizers (not specified) daily or near daily increased the risk of developing MOH by 5.2-fold and that using analgesics for any condition increased the risk by 3.0-fold [54]. The time to onset of MOH varies with medication type as well; it is shorter for triptans, opioids, and combination agents than for simple analgesics [55].

Excessive acute headache medication use may also be driven by lack of use or suboptimal use of preventive treatment, including medications, non-pharmacologic therapies, and lifestyle modifications. When preventive medication is effective, it reduces the number of MHDs; however, if preventive medication is not optimized, headache frequency may remain high. In this context, high MHD frequency and excessive use of acute treatments may contribute to increasing MHD frequency. The longitudinal LUMINA study found that using preventive medications at baseline was an independent predictor of migraine-day frequency at follow-up (mean ± standard deviation: 93 ± 30 weeks post-baseline), with a > 5.1-fold increased risk of having an increased frequency of migraine days at follow-up [47]; these results suggest that preventive medication available at the time of the LUMINA study may have been suboptimal.

### Comorbidities

Numerous comorbidities are associated with higher MHD frequencies in cross-sectional studies, and several comorbidities have been linked to an increased risk of new-onset CM or CDH in longitudinal studies, when considered one at a time or as part of a constellation of comorbidities.

#### Comorbidities associated with higher MHD frequency

Several comorbidities are associated with higher headache frequencies in cross-sectional studies. These comorbidities include psychiatric disorders, metabolism-related conditions, chronic pain conditions, traumatic head injury, poor sleep quality and sleep disturbances, and respiratory conditions.

The AMPP, Migraine in America Symptoms and Treatment (MAST), and American Registry for Migraine Research studies found that rates of comorbid depression and anxiety increased with higher MHD frequency [4, 25, 27]. In the AMPP study sample, rates of depression increased from 23.5% in participants with 0–3 MHDs to 39.5% in participants with ≥ 15 MHDs, rates of severe depression from 2.9 to 9.0%, respectively, and rates of nervousness/anxiety from 17.4 to 29.2%, respectively [4]. In MAST, the odds ratio of having comorbid anxiety or depression significantly increased as MHD frequency increased [27]: relative to individuals with 1–4 MHDs, the odds of anxiety increased 1.4-fold for those with 5–9 MHDs and 2.3-fold for those with ≥ 21 MHDs; the odds of depression increased 1.4-fold in those with 5–9 MHDs and 2.4-fold in those with ≥ 21 MHDs.

Chronic pain conditions (e.g., fibromyalgia, back/neck pain, arthritis) are more common in those with more frequent migraine/headache days [54, 56, 57]. For example, a cross-sectional survey of headache clinic patients with migraine found that those who reported ≥ 4 pain comorbidities were more likely to be diagnosed with “transformed migraine” (or CM) than those who had ≤ 3 pain comorbidities [57]. Compared with those who had no pain comorbidities, individuals with pain conditions were significantly more likely to be diagnosed with CM or with continuous daily headache [57]. In the MAST study, relative to individuals with 1–4 MHDs, subgroups with ≥ 5 MHDs had significantly higher odds of having comorbid arthritis (type unspecified), osteoarthritis, or rheumatoid arthritis [27]; individuals experiencing ≥ 21 MHDs had a 2.6-fold increased likelihood of having arthritis (type unknown). The AMPP study found that individuals with high-frequency EM were 77% more likely to have chronic pain and 25% more likely to have rheumatoid arthritis/osteoarthritis compared with those with low-frequency or medium-frequency EM [4]; individuals with CM were 35% more likely to have chronic pain and 44% more likely to have rheumatoid arthritis/osteoarthritis than participants with high-frequency EM.

Metabolism-related comorbidities are highly prevalent in advanced disease states, including obesity and metabolic syndrome. A meta-analysis found an increased risk of CM in overweight individuals (body mass index [BMI] 25 to < 30 kg/m^2^) and an even greater risk of CM in obese individuals (BMI ≥ 30 kg/m^2^) compared with those of normal weight [58]. The AMPP study also found higher rates of obesity as MHD frequency increased; however, the odds ratios were not significant between MHD groups in adjusted models [4]. In a cross-sectional, clinic-based study of women with migraine, metabolic syndrome was associated with CM [59]. In addition, when CM was comorbid with MOH, the risk of metabolic syndrome significantly increased and MOH was, in particular, associated with abdominal obesity and hypertension components of metabolic syndrome [59]. A clinic-based study found that insulin resistance, a major component of metabolic syndrome, is more prevalent in women with CM compared with women with EM, and that having comorbid obesity alongside insulin resistance further increased the risk of CM [60].

Poor sleep quality and sleep disturbances, such as snoring, insomnia, and sleep apnea, are associated with headache and migraine [27, 44, 61–63]. The MAST study found that the odds ratio of having comorbid insomnia significantly increased as MHD frequency increased [27]; relative to individuals with 1–4 MHDs, the odds of insomnia increased 1.6-fold in those with 5–9 MHDs and 2.6-fold in those with ≥ 21 MHDs. In the CaMEO study, more respondents with CM than EM were at “high risk” for having sleep apnea (52% vs 37%, respectively) based on a screener; they also had higher rates of self-reported sleep apnea (14% vs 10%) [63]. This analysis also found that respondents with CM had poorer sleep quality than those with EM. A population-based study in major metropolitan areas found that, compared with controls with episode headache, participants with CDH had 2.9-times more likely to snore, even after controlling for factors associated with sleep-disordered breathing, and there was evidence of a dose–response relationship [61]. One study using diaries found that excessive sleep and restless sleep were two of eight significant triggers for migraine initiation in the study population (11% for each) [64].

Respiratory conditions such as asthma and chronic rhinitis have also been associated with increased headache frequency and headache-related disability [51, 65]. The MAST study found significant odds ratios of having comorbid asthma in higher MHD frequencies [27]; relative to individuals with 1–4 MHDs, the odds of asthma ranged from 10% higher with 5–9 MHDs to 90% higher with ≥ 21 MHDs. The AMPP study found that individuals with high-frequency EM were 34% more likely to have asthma and 65% more likely to have chronic bronchitis compared with those with low-frequency or medium-frequency EM [4].

#### Comorbidities associated with new-onset CM or CDH

Psychiatric comorbidities and symptoms (more specifically, depression and anxiety) are associated with an increased risk of CM and MOH [27, 43, 66, 67]. In the AMPP cohort, depression was a significant predictor of CM onset, and the risk of CM transition increased with the severity of depression [66]. Anxiety was also associated with increased risk of CM [66]. The LUMINA cohort study found that having a lifetime history of depression at baseline was associated with an eightfold increased risk of having an increase in migraine-day frequency over almost 2 years of follow-up [47]. Anxiety was associated with a twofold increased risk while depression was associated with a 2.6-fold increased risk of developing MOH in the HUNT cohort study [54]. Additionally, the risk of MOH increased 4.7-fold in participants with musculoskeletal pain and gastrointestinal complaints in combination with anxiety and depression [54].

A handful of longitudinal studies have found an increased risk of new-onset CM in the presence of other comorbidities. The longitudinal Frequent Headache Epidemiology Study found that the odds of progression to CDH were significantly higher in individuals with head and neck injuries, with the lifetime risk increasing with increased number of injuries [68]. In participants with EM in the CaMEO study, the odds of CM onset over 3 months were increased by 22% for each individual extra-cephalic pain comorbidity, after accounting for potentially confounding or mediating factors [69]. A population-based study in several major metropolitan areas in the US found that, compared with controls, participants with EM and BMI ≥ 30 kg/m^2^ at baseline had 5.3-fold increased risk of incident CDH at follow-up after controlling for BMI and baseline headache frequency [44]. The HUNT studies found a positive association between insomnia and new-onset chronic tension-type headache and CM [62]. Lastly, the risk of new-onset CM at 1-year follow-up was more than twofold higher in those with asthma compared with those without asthma in the AMPP study [51].

Using latent class modeling with longitudinal CaMEO cohort data, the onset of CM was analyzed in individuals with EM categorized into eight comorbidity classes defined using latent class modeling. The eight groups are: most comorbidities; respiratory/psychiatric; respiratory/pain; respiratory; psychiatric; cardiovascular; pain; and fewest comorbidities) [67]. Relative to the “fewest comorbidities” class, the study showed that each comorbidity class was significantly associated with increased risk of progression from EM to CM (hazard ratios of 1.5–5.3 after controlling for demographics) [67]. The “most comorbidities” group had the largest hazard ratio (5.3 [95% confidence interval: 3.89, 7.33]), followed by the group with a combination of respiratory and pain comorbidities (3.6 [2.67, 4.98]) and the group with psychiatric disorders (2.4 [1.77, 3.28]) [67]. The risk of progression to CM attributed to comorbidities was attenuated but remained significant after adjusting for headache-related features [67].

### Lifestyle and exogenous factors

Lifestyle and exogenous factors, such as stressful events, adverse childhood experiences, unhealthy diet, poor sleep, and lack of exercise, may predispose to or trigger an individual migraine attack, and frequent exposure to such factors may lead to migraine disease progression [70].

Stressful events, including major life events, adverse childhood experiences, daily hassles, and ongoing financial constraints, can influence the onset of chronic headache. The community-based Frequent Headache Epidemiology Study assessed the relationship between six categories of major life events or changes (related to work, relationships, children, residence, deaths, and other self-defined changes such as financial, illness, and abuse) and the onset of CDH [71]; after adjustment for age, sex, headache type, and year of the event, the odds of CDH increased by 20% with each additional antecedent event. Ongoing financial constraints are also an important risk factor for CM, as discussed in the section on demographic risk factors.

Cross-sectional analyses in nationally representative populations of adults (from the United States or Canada, mean ages ranging from 29 to 50 years) have shown an independent relationship of migraine and multiple types of adverse childhood experiences, including emotional abuse and witnessing domestic violence, even without physical contact [72–74]. Each study showed a dose–response effect, with increasing numbers of abuse types associated with rising odds of migraine. The AMPP study showed each maltreatment type was associated with higher MHDs in people with migraine compared with those with tension-type headache [74]. In a cross-sectional, multicenter, headache clinic-based sample, childhood emotional abuse was associated with a 77% increase in odds of CM and an 89% increase in odds of transformation from EM to CM, after adjustment for sociodemographic factors, anxiety, and depression [75]. Other cross-sectional studies further demonstrated that emotional abuse was linked to migraine-associated allodynia and to increased total number of comorbid pain conditions, which in turn were linked together in a graded fashion [57, 76]. These results are supported by the recent cross-sectional observation that prior abuse is associated with an increase in migraine-related hypersensitivity symptoms [77].

Dietary habits are also associated with migraine progression. One cross-sectional study evaluating the effect of an inflammatory diet pattern on migraine found that individuals with > 20 MHDs had higher Dietary Inflammatory Index values compared with individuals with < 10 MHDs [78], and another cross-sectional study found that individuals who had Dietary Inflammatory Index scores in the upper tertile of the study experienced greater headache frequency than individuals in the lowest tertile [79].

Poor hydration is linked to worse migraine symptoms. One cross-sectional study observed a negative dose response between migraine characteristics and daily water intake. Women who drank less water experienced more migraine disability, higher headache frequency, greater pain intensity, and longer duration of headaches [80].

The relationship between caffeine intake and migraine is complicated, but there are data to suggest that high caffeine intake in the past, or changes in intake, are associated with migraine attacks [81–83]. A case–control study found that individuals with CDH were approximately 50% more likely than those with episodic headache to have been high consumers of dietary and medicinal caffeine prior to CDH onset than high consumers after CDH onset, with high consumption defined as being in the upper quartile of caffeine consumption (roughly three 8-oz cups of caffeinated coffee) or using caffeine-containing non-prescription analgesics as their preferred headache treatment [81]. Abrupt change in usual caffeine intake in general is also a risk factor for migraine attacks, but although caffeine overuse has been suggested to lead to progression, the contribution of current caffeine use to migraine progression from EM to CM is unclear [82, 83]. Results from the longitudinal HUNT studies study indicate that daily caffeine use of > 540 mg compared with use of ≤ 240 mg results in a 1.4-fold increase in risk of MOH [54].

In the HUNT study, physical inactivity was associated with a 2.7-fold increased risk of MOH but was not associated with CDH without medication overuse [54]. In alignment with the HUNT surveys, the cross-sectional Danish National Health Survey found that chronic headache was associated with low physical health composite scores after controlling for possible confounding variables [84].

Smoking tobacco is also associated with migraine disease progression. In the HUNT study, smoking was associated with a 1.8-fold increased risk of MOH but not with CDH without medication overuse [54]. A separate cross-sectional study also reported that daily smoking was significantly associated with MOH in both men and women [85].

Exposure to triggers specific to individuals may increase attacks. One longitudinal study examining triggers found that factor profiles were unique and highly individual in 85% of those who had at least one factor associated with their migraine attacks, highlighting the need to identify personal triggers in migraine prevention [64]. A prospective observational study detected distinct relationships between migraine and stress using diary entries (high stress, a sudden decrease in stress, and no relationship to stress), emphasizing individual responses to triggers [86]. Another prospective observational study using diary entries examined the effect of an improbable event on migraine occurrence [82]. For example, a person who rarely has stressful events would be more likely to experience a migraine attack if they suddenly had 10 stressful events, compared with someone who usually experiences 10 stressful events per day.

### Demographic factors

Being female is a risk factor for migraine and is associated with a greater risk of having CM. The AMPP study reported that among individuals with migraine, 1.29% of women versus 0.48% of men met the criteria for CM [87]; women also experienced severe headache-related disability more often than men. In the HUNT studies, there was a 1.9-fold higher risk of women developing MOH compared to men [54].

Hormonal status can also influence migraine frequency and prevalence. For instance, a cross-sectional observational study found that perimenopausal women are more likely to experience a higher frequency of migraine attacks than premenopausal women, suggesting that fluctuations in hormonal status in women can increase migraine frequency [88]. In men, lower levels of testosterone are observed in individuals with chronic migraine [89]. In one study of women receiving testosterone pellet implants, migraine attack intensity was reduced, and 74% of the participants experienced no migraine attacks by their second dose [90].

In a cross-sectional analysis of the AMPP study, there was a strong inverse correlation between household income and CM, with the highest income levels having the lowest prevalence of CM [87]. The adjusted prevalence of CM among women/girls > 12 years of age in households with annual incomes below $22,500 was 2.7% but only 0.52% for women/girls in households with annual incomes above $90,000 per year [87]. Similarly, in a longitudinal study among young adolescents, lower household economic status (defined as “below average” or “poor”) was associated with a ≥ twofold increased risk of CM [91].

The HUNT study found that low level of education attainment was associated with a 1.7-fold and 1.9-fold higher risk developing CDH and MOH, respectively, compared to high educational attainment [54].

## Potential protective actions to attempt to prevent progression

The identification of modifiable predictors of migraine progression is an important first step towards developing targeted interventions that protect against progression. While associations between modifiable risk factors and disease progression suggest the possibility of intervention to prevent progression, this has yet to be rigorously confirmed conclusively in clinical trials for most risk factors.

Protective actions for the five domains of risk factors include acute and preventive treatment optimization (including pharmacologic and non-pharmacologic therapies), comorbidity management when possible, and lifestyle management (Table 1). The recommendations provided here are based on various levels of published evidence and expert opinion. For some interventions, there is published literature supporting the recommendations; for others, actions are less substantiated by the literature but are guided by best clinical practice based on expert opinion.

When assessing a patient’s risk profile for migraine progression, it is important to include the patient as a partner in developing a management approach. Clinicians should consider the evidence supporting that the intervention may modify progression risk coupled with the patient’s willingness, motivation, and expressed interest or agreement in participating. Thus, a patient-centered approach that emphasizes both physical and psychological well-being can improve treatment adherence, outcomes, and patient satisfaction [92–94]. Multidisciplinary support including non-pharmacologic therapies for those with more frequent migraine should be offered to support lifestyle and comorbidity management when appropriate as well as for the behavioral management of migraine.

### Protective actions for migraine disease and treatment-related risk factors

There are several protective actions that can be applied to many risk factors associated with migraine burden and treatment, which are likely to reduce the risk of migraine disease progression. Acute treatment optimization, addressing acute medication overuse, and considering pharmacologic and non-pharmacologic preventive therapies when appropriate should be prioritized, as suboptimal acute treatment is a strong risk factor for migraine disease progression as detailed earlier. However, a longitudinal relationship has not yet been demonstrated conclusively between improvements in optimization and reduction in risk of new-onset CM in a clinical trial.

Optimizing acute and preventive treatment using both pharmacologic and non-pharmacologic options can address risk factors—including high numbers of MHDs (≥ 5 for moderate risk and ≥ 10 MHDs for high risk of progression)—alleviate or prevent allodynia, prevent persistent/ frequent nausea by appropriately treating migraine, and more effectively prevent or treat severe migraine symptoms [1, 21, 24, 95–103].

The mTOQ is commonly used to evaluate acute headache medication optimization. The questions address relief of migraine pain within 2 h, sustained headache freedom for the next 24 h, rapid return to normal activities, and good medication tolerance [29]. When initiating and adjusting treatment regimens, such goals should be kept in mind. However, a continuum of acute headache medication optimization should also be offered to further improve HRQoL, such as when an intervention reaches a response plateau, or an additional new intervention could further improve symptoms even after achieving mTOQ relief milestones. For patients with inadequate response to their current regimen, individualization of therapy should be considered (e.g., switching, titrating, combining, multidisciplinary), while also including the patient as a partner in developing a management approach and considering which changes will have the greatest impact on their life. This could include considering nonpharmacological approaches, the dose or route of administration, adding or switching to another medication, adding a preventive medication, or optimizing medication plan adherence.

Medication overuse is a significant risk factor for migraine progression, not only for transforming from EM to CM, but also for developing MOH [22, 47, 52]. Patients should be prescribed appropriate acute medication, but also should be educated on the risks of increased use. Expert opinion suggests limiting acute medication to < 10 days/month. In this way, patients will be mindful of their acute medication use and are more likely to notice an increased need for medication, a sign that they should consult with their healthcare provider [22, 47, 52]. Patient education and building a communicative relationship can also be an effective tool in acute medication optimization.

Preventive treatments may reduce the risk of progression by preventing the occurrence of migraine attacks and reducing acute medication use. The INTREPID study was a randomized trial designed to test the hypothesis that topiramate prevented the transition from EM to CDH in persons with migraine and 9–14 monthly headache days. Although rates of transition to CDH were lower with topiramate treatment than with placebo, differences were not statistically significant, perhaps because the study was terminated prematurely due to loss of funding. There is good evidence that several preventive treatments can reduce MHDs and move patients from CM to EM, although evidence for preventing the transition from EM to CM is not robust [5, 104–111]. There is also good evidence that preventive treatments can reduce the need for acute medication use in patients with medication overuse or MOH; however, preventing the onset of either with preventive treatments has yet to be studied fully [105, 112, 113]. Preventive medication can also improve overall acute medication optimization in patients, keeping in mind that it is important for patients to take preventive medication as prescribed [114]. In addition to preventive medication, non-pharmacologic preventive options may work well for some patients.

Suggesting the use of a diary to track the frequency, intensity, and nature of these risk factors will allow the patient and the clinician to monitor the progression of the disease over time, to quickly identify if the disease is progressing, or to discern if changes need to be made to the patient’s treatment plan. Clinicians can also educate their patients on healthy lifestyle choices and how to implement change. One specific protective action with moderate evidence for individuals experiencing high numbers of MHDs (≥ 5 for moderate risk and ≥ 10 for high risk) is progressive muscle relaxation. A longitudinal study of this technique demonstrated a 41% reduction in the number of migraine attacks and a 43% reduction in migraine days [115].

Integrating non-pharmacologic approaches such as regular exercise, neuromodulation, biofeedback, cognitive behavior therapy (CBT), relaxation therapies, mindfulness-based therapies, and acceptance and commitment therapy may also have the potential to prevent migraine progression through multiple means, including reducing headache days, reducing associated disability, possibly reducing acute medication overuse, and improving resilience, coping skills, and behavioral management strategies [1, 95]. Biobehavioral and neuromodulation therapies are listed in the American Headache Society consensus statement for acute (neuromodulation) and/or preventive (neuromodulation and behavioral therapies) treatment of migraine [1, 116, 117]. For example, CBT, biofeedback, and relaxation training have been validated for prevention to improve measures of severity (including intensity, duration, and frequency) of migraine and tension-type attacks, as well as comorbid depression, anxiety, and HRQoL in a longitudinal randomized controlled trial [118]. There are a variety of neuromodulation options for both acute and preventive treatment of migraine, such as remote electrical neuromodulation, noninvasive vagal nerve stimulation, external trigeminal nerve stimulation, single-pulse transcranial magnetic stimulation, and supraorbital transcutaneous stimulation, that have been established as safe and effective preventive therapies for migraine [96–101].

### Protective actions for comorbidity risk factors

Comorbidity management is an important component of a migraine management strategy and should be guided by a collaborative patient‒clinician decision-making paradigm. Referral to a specialist and multidisciplinary providers may be appropriate depending upon comorbidities and patient variables. While there are several comorbidities associated with migraine progression, there is limited direct evidence to suggest that treating comorbidities will decrease progression. Nevertheless, increased attention to the management of the most significant known comorbidities associated with migraine progression (e.g., depression, anxiety, chronic pain, history of head or neck injury, metabolism-related comorbidities, sleep disturbances, allergic rhinitis, asthma, female sex, hormones) represent a reasonable approach to mitigating the risk of progression and should not be overlooked.

Education on the risks of medication overuse should be offered routinely to all patients with migraine but should be based on individual circumstances [119]. Motivational interviewing and reinforcement of positive behaviors can affect behavioral changes and promote positive relationships [120]. Such initiatives are designed to stimulate patients’ desire to change and give them the confidence to do so, but they can be challenging to implement, as this requires an entirely different perspective on the nature of the clinician’s role.

### Protective actions for lifestyle/exogenous risk factors

Although evidence demonstrates that lifestyle and exogenous factors are related to increasing MHDs, there is little research on potential interventions. These are the areas where expert opinion offers supportive insight. While the protective actions below are described from a migraine standpoint, lifestyle and exogenous factors should be attended to regardless of their possible relation to migraine disease and its progression.

Stress in many forms influences migraine, including stress from adverse childhood events, stressful life events, ongoing financial constraints, and previous assault. Individuals with stress could benefit from approaches such as stress management, CBT, biofeedback, relaxation training, exercise, social support, or fostering resilience to reduce the frequency of migraine based on their individual needs. Several of these approaches (CBT, biofeedback, relaxation training) already have evidence supporting their use for migraine management, but not specifically within the context of stress [1, 95, 116, 117].

Poor dietary habits and lack of hydration are also associated with elevated MHDs in cross-sectional studies [78, 79]. Possible interventions could include providing education and resources on how to eat regular, balanced meals and recommendations for staying hydrated. Accommodations, such as a note to allow more access to water during work hours, could also be beneficial.

Former and current high caffeine intake is associated with migraine progression [54, 81–83]. Due to this, expert opinion is to limit caffeine intake to no more than 2 caffeinated beverages daily.

Physical inactivity is another lifestyle factor linked to increased frequency of migraine [121]. There is a lack of data on the prevention of disease progression using physical activity as a treatment, but expert opinion recommends increasing physical activity as a preventive measure, as aerobic exercise affects migraine frequency [121].

Poor sleep habits and hygiene practices are also risk factors for disease progression separate from specific sleep disorders such as sleep apnea or insomnia. Literature suggests that addressing sleep hygiene can revert patients with CM back to EM and decrease headache intensity [122]. Encouraging patients to set a consistent bedtime and waking time that allows for 8 h in the bed; eliminate stimulating activities such as television, reading, or music in bed; use visualization techniques to reduce time to sleep onset; set the last meal of the day to be > 4 h before bedtime; limit fluid intake within 2 h before bedtime; and avoid naps during the day has been shown to be beneficial in reverting migraine progression and decreasing headache intensity [122]. Further, some have recommended behavioral sleep regulation, management for sleep apnea, and CBT for insomnia as complementary measures to standard practice [123].

Tobacco cessation may be a possible intervention for migraine progression as smoking is associated with MOH [54, 85].

Personal triggers are likely to contribute to migraine progression if an individual is unaware of their migraine triggers. As mentioned previously, repeated and prolonged activation of the trigeminovascular system and its central connections may lead to a lowered threshold for initiating migraine attacks [21]. An individual repeatedly exposing themselves to an unknown migraine trigger could result in persistent activation and central sensitization. It is recommended that patients keep a headache diary for the purpose of identifying triggers or trigger combinations unique to them, as retrospective recall can over- or underestimate the contribution of a perceived trigger [124].

Therapies are available in a multitude of formats to improve access to biobehavioral and educational assistance to aid in initiating and successfully implementing lifestyle changes, including changes to improve mental well-being. They may include telehealth, group-based formats, web-based formats, app-based programs, wearables, bibliotherapy, and other affordable and accessible options. These approaches can include interactive digital modules on migraine-specific knowledge, emotional coping, communication skills, migraine self-management skills, relaxation training, and medication safety, or using a combination of healthcare professional interactions and digital technology [125, 126]. One systematic review found that nearly 70% of the evaluated studies examining the efficacy of digital self-management techniques demonstrated improvements in headache-related outcomes [127].

## Conclusions

**Fig. 1:**
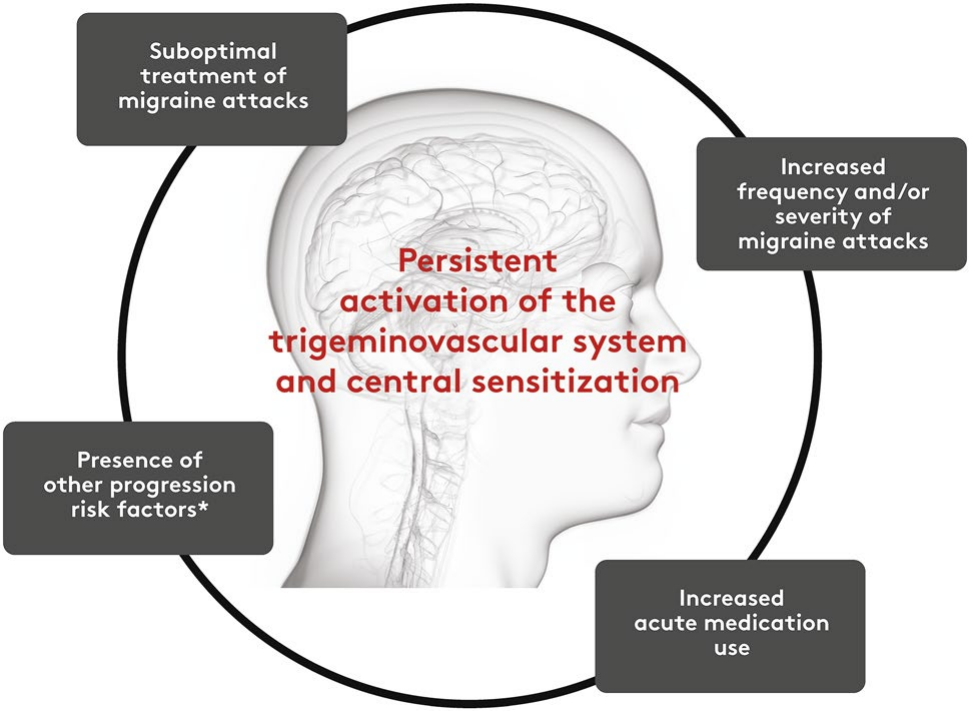
A model of migraine disease progression. *See Table 1

This review aims to provide clinicians with information to identify migraine progression, knowledge of risk factors for migraine progression, and potential mitigation strategies supported by research and expert opinion. A proposed model of migraine disease progression is presented in Fig. 1. Increased migraine frequency and ineffective or suboptimal use of acute headache medication are major contributors to migraine disease progression. This supports the hypothesis that migraine progression is associated with risk factors and changes in pathophysiology in tandem. Therefore, it may be important to manage migraine carefully with a personalized, patient-centered approach including education, lifestyle enhancements, and optimized pharmacologic and non-pharmacologic therapies as appropriate to avoid new-onset progression or further disease progression.

Key risk factors for migraine progression were categorized into 5 broad domains: migraine disease characteristics, treatment-related factors, comorbidities, lifestyle/exogenous factors, and demographic factors. Risk factors for migraine progression with a good level of evidence were numerous and included MHD frequency, suboptimal acute headache treatment, cutaneous allodynia, medication overuse, psychiatric symptoms, other chronic pain, metabolism-related comorbidities, sleep disturbances, respiratory conditions, former and current high caffeine intake, physical inactivity, tobacco use, and personal triggers as risk factors for migraine progression. Risk factors with moderate evidence include ≥ 5 MHDs (moderate risk) or ≥ 10 MHDs (high risk), persistent or frequent nausea, former head or neck injury, female sex, hormonal status, stress, poor nutrition, poor hydration, low level of education attainment, and poor sleep quality and duration. Further research is needed to develop a statistical model that weights risk factors predicting migraine disease progression; a model as such could be used to identify patients most at risk for progression and to guide prioritization of addressing risk factors to prevent or revert progression.

Actions that protect against migraine progression seldom appear in published literature and are therefore primarily based on expert opinion; however, there is ample literature suggesting that optimizing acute headache medication is effective at reducing progression risk [1, 95–101]. The implementation of interventions should be patient-centered and focus on interventions that are the most meaningful and feasible.

Knowledge of migraine disease progression, risk factors, and use of protective actions will allow clinicians to identify at-risk patients and to initiate interventions designed to minimize risk of progression.

### Supplementary Information

Below is the link to the electronic supplementary material.

Supplementary file1 (PDF 120 kb)

Supplementary file2 (PDF 107 kb)

## Data Availability

As a narrative review article, published data are available in the original articles and summarized here.

## Abbreviations

AMPP: American Migraine Prevalence and Prevention
BMI: Body mass index
CaMEO: Chronic migraine epidemiology and outcomes
CBT: Cognitive behavior therapy
CDH: Chronic daily headache
CM: Chronic migraine
EM: Episodic migraine
HRQoL: Health-related quality of life
HUNT: Nord-Trøndelag Health Study
ICHD-3: International Classification of Headache Disorders, 3rd edition
LUMINA: Leiden University Migraine Neuro-Analysis
MAST: Migraine in America Symptoms and Treatment
MHD: Monthly headache day
MOH: Medication-overuse headache
mTOQ: Migraine Treatment Optimization Questionnaire
NSAID: Nonsteroidal anti-inflammatory drug
