# Supplementary figures and images for: Risk factors for migraine disease progression: a narrative review for a patient-centered approach
Source: J Neurol. 2023 Aug 24;270(12):5692–710. doi: 10.1007/s00415-023-11880-2 (PMC10632231; doi:10.1007/s00415-023-11880-2)

## Supplementary Figure 1. Visual abstract of migraine progression

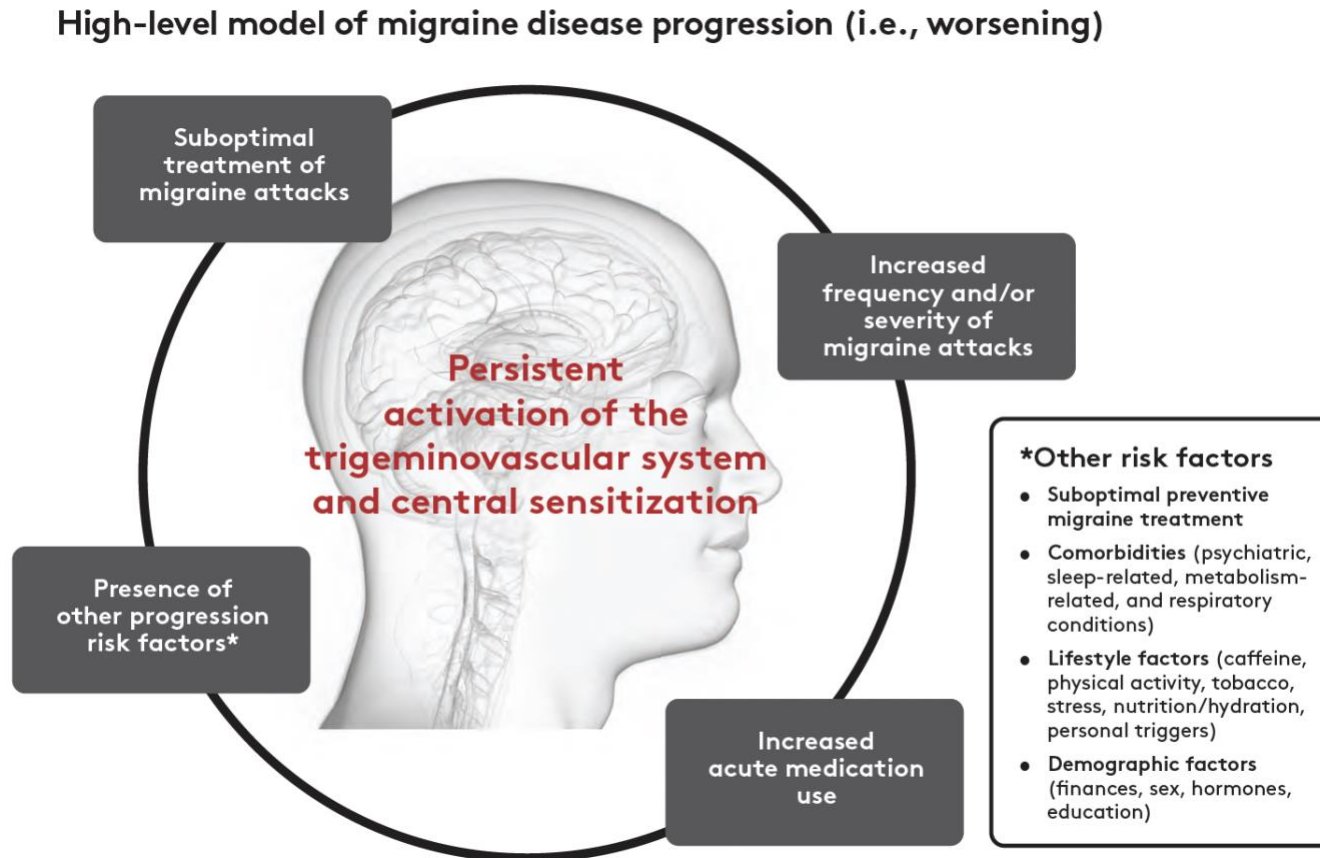

Supplement: Supplementary file 1 — Supplementary file1 (PDF 120 kb) [file 415_2023_11880_MOESM1_ESM.pdf]
