# Supplementary material for: Risk factors for migraine disease progression: a narrative review for a patient-centered approach
Source: J Neurol. 2023 Aug 24;270(12):5692–710. doi: 10.1007/s00415-023-11880-2 (PMC10632231; doi:10.1007/s00415-023-11880-2)
Supplement: Supplementary file 2 — Supplementary file2 (PDF 107 kb) [file 415_2023_11880_MOESM2_ESM.pdf]

Lipton RB, Buse DC, Nahas SJ, et al. Risk factors for migraine disease progression: A narrative review for a patient-centered approach. *Journal of Neurology*. 2023.
Correspondence: Hans-Christoph Diener, Institute for Medical Informatics, Biometry and Epidemiology, Medical Faculty of the University Duisburg-Essen, Germany, hans.diener@uk-essen.de

Supplementary Table 1. Printable summary of key risk factors of migraine disease progression and possible protective actions

	Risk factors	Level of evidence	Possible protective actions	Level of evidence
**Migraine disease characteristics**	• *Any migraine disease characteristic risk factor*		• Track attack frequency, severity, and treatment use through a diary or app<br>• Optimize acute and preventive treatment using both pharmacologic and nonpharmacologic options<br>• Educate patients on healthy lifestyle choices and how to implement change	O<br>↑<br>O
	• ≥5 MHDs (moderate risk) or ≥10 MHDs (high risk)	↔	• Progressive muscle relaxation	↔
	• Cutaneous allodynia	↑		
	• Persistent, frequent nausea	↔		
**Suboptimal treatment**	• *Any treatment-related factor*		• Use nonpharmacologic approaches such as neuromodulation and biobehavioral therapies<br>• Change dose or route of administration<br>• Add or switch to another acute or preventive medication<br>• Add preventive therapies<br>• Optimize adherence	↔<br>O<br>O<br>O<br>O
	• Suboptimal acute treatment	↑	• Optimize acute migraine medication<br>• Educate patients about their acute migraine medication	O<br>O
	• Acute medication overuse	↑	• Educate patients about appropriate exposure to acute migraine medication and risks of high use<br>• Limit acute medication use to <8 days/month	O<br>O
	• Preventive medication not satisfactorily effective/tolerated	O	• Optimize preventive medication<br>• Aim for treatment to reduce days of moderate or severe pain to <4 days per month<br>• Take preventive medication as prescribed<br>• Consider adding nonpharmacologic preventive options	↑<br>O<br>O<br>O
**Comorbidities**	• *Any comorbidity*		• Identify common comorbidities and educate patients on comorbid conditions<br>• Optimize assessment and treatment of comorbid conditions where appropriate<br>• Refer for treatment when appropriate<br>• Teach self-management skills and strategies when appropriate	O<br>O<br>O<br>O
	• Psychiatric symptoms, especially depression and anxiety	↑	• Assess, monitor, treat, or refer to mental health professionals for pharmacologic and/or behavioral treatment	O
	• Other (non-headache) chronic pain conditions	↑		
	• Head and neck injury, TBI	↔	• Physical therapy	O

Lipton RB, Buse DC, Nahas SJ, et al. Risk factors for migraine disease progression: A narrative review for a patient-centered approach. *Journal of Neurology*. 2023.
Correspondence: Hans-Christoph Diener, Institute for Medical Informatics, Biometry and Epidemiology, Medical Faculty of the University Duisburg-Essen, Germany, hans.diener@uk-essen.de

	Risk factors	Level of evidence	Possible protective actions	Level of evidence
	• Metabolism-related comorbidities (metabolic syndrome, insulin resistance, overweight/underweight)	↑	• Exercise/physical activity<br>• Diet<br>• Optimized disease management	O<br>O<br>O
	• Sleep disturbances, including insomnia, snoring, and restless leg syndrome	↑	• Healthy sleep hygiene and practices<br>• Targeted behavioral sleep intervention<br>• May need a sleep study (obstructive sleep apnea)<br>• May need CBTi (insomnia)	O<br>↔<br>↔<br>↔
	• Respiratory conditions such as allergic rhinitis and asthma	↑		
	• Multiple other comorbidities	↔		
**Lifestyle and exogenous factors**	• *Any lifestyle or exogenous risk factor*		• Self-management strategies and programs	↔
	• Stress, including adverse childhood experiences, stressful life events, previous assault	↔	• Refer for therapy and support<br>• Stress management, CBT, biofeedback, relaxation training, exercise, social support<br>• Foster resilience	O<br>O<br>O
	• Poor nutrition	↔	• Eat regular, balanced meals	O
	• Poor hydration	↔	• Stay hydrated with water and other hydrating liquids<br>• Provide access resources such as a note for work to allow more access to water	O<br>O
	• Former and current high caffeine intake	↑	• Reduce caffeine intake to no more than 2 caffeinated beverages a day	O
	• Physical inactivity	↑	• Regular exercise/physical activity	↔
	• Poor sleep quality and duration	↔	• Healthy sleep hygiene and practices	↔
	• Smoking tobacco	↑	• Tobacco cessation	O
	• Exposure to personal triggers	↑	• Keep a headache diary to identify triggers	↔
**Demographic factors**	• Female sex	↔		
	• Hormonal status	↔		
	• Low level of education attainment	↔		
	• Ongoing financial constraints	↑		

Level of evidence (for the risk factor association with progression, or that addressing the risk factor reduces progression risk): ↑ = good evidence; ↔ = moderate evidence; O = expert opinion.

CBT, cognitive behavioral therapy; CBTi, cognitive behavioral therapy for insomnia; MHDs, monthly headache days; TBI, traumatic brain injury.
